# Alcohol Research and eHealth Technology

**DOI:** 10.35946/arcr.v36.1.01

**Published:** 2014

**Authors:** Tammy Chung, Michael Hilton

**Affiliations:** Tammy Chung, Ph.D., is associate professor of psychiatry and epidemiology in the Department of Psychiatry, University of Pittsburgh, Pittsburgh, Pennsylvania. Michael Hilton, Ph.D., is deputy director of the Division of Epidemiology & Prevention Research, National Institute on Alcohol Abuse and Alcoholism, Bethesda, Maryland.

The rapid advance of electronic technology holds the promise for revolutionary improvements in conducting research on alcohol use disorders as well as innovative methods for prevention and treatment. This issue of *Alcohol Research: Current Reviews* reports on the state of the science and future directions in electronic health (eHealth) technologies and their potential impact on alcohol epidemiology, prevention, and treatment. As an evolving transdisciplinary field, eHealth is poised to transform theories of behavioral change and models of behavioral health care. eHealth brings real-time, in-the-moment monitoring of bodily and cognitive states and enables us to deliver personalized “just-in-time” interventions.

eHealth includes mobile health (mHealth) and covers, for example, devices (e.g., computers, smart phones, tablets) for monitoring and delivery of information and interventions; sensors (e.g., geographical positioning system [GPS], accelerometers, glucose and heart rate monitors, transdermal alcohol sensors); Internet and social media (e.g., text messaging, Twitter, Facebook, blogs, chat rooms, Internet support groups, Instagram); computerized interventions (e.g., see the articles by Bickel and by Carroll); eHealth “apps” (Aguilera and Muench 2012); and the integration of multiple technology types in seamlessly coordinated health care platforms (e.g., see the article by Quanbeck).

The lexicon describing these new technologies also is evolving (Eysenbach 2001; Oh et al. 2005), such that the term “digital health technologies,” which subsumes eHealth and mHealth and encompasses health information technology and personal genomics (Topol 2012), is now becoming more mainstream.

At the National Institutes of Health (NIH), a variety of activities reflect the NIH’s belief that eHealth will become a core component of personalized medicine. Specifically, the NIH Wireless Medical Technologies Working Group coordinates mHealth activities across Institutes to accelerate the translation of advances in eHealth to personalized care. The NIH also coordinates the mHealth Summit, an annual meeting that facilitates collaboration on wireless health technology development and application among academia, the private sector, and health care providers (Nilsen et al. 2013). At NIAAA, research initiatives include funding opportunities, in conjunction with Collaborative Research on Addiction (CRAN), on the use of social media to address alcohol and other substance use and the use of ecological momentary assessment (EMA) to study mechanisms of behavior change in alcohol treatment.

Reviews in this issue highlight how eHealth technologies are changing our thinking about public health surveillance and the collection of data for alcohol research (see the articles by Beckjord and by Freisthler) and beginning to transform clinical practice across the continuum of care (see the articles by Cronce, by Harris, and by Quanbeck). With such extensive access to computers, mobile devices, and the Internet in the United States (Duggan 2013), eHealth has the capacity for broad reach, including traditionally underserved populations (e.g., rural communities, racial/ ethnic minority groups) (Gibbons et al. 2011; Marsch 2012). Internet-or computer-delivered education, screening, and intervention can offer a cost-effective means of reducing barriers to help-seeking through on-demand, round-the-clock access to information and services (Strecher 2007). The reviews in this issue, written by leaders in the field, discuss the implications of eHealth technologies for refining the measurement and monitoring of alcohol use, revising theories of behavioral change, and restructuring models of health care delivery.

Real-time measurement of behaviors as they occur in the natural environment through self-report, sensors (e.g., GPS location, alcohol wrist sensor), and social media exchanges (e.g., Twitter, Facebook updates) has dramatically increased the frequency of measurement and the types of data that can be collected simultaneously (Wang et al. 2014). Applied to alcohol research, EMA has been used to study event-level risk factors for heavy drinking and adverse consequences (see the article by Wray). EMA combined with biomonitoring (e.g., alcohol wrist sensors) has been used to improve the accuracy of alcohol consumption surveys and to monitor treatment outcome (see the article by Greenfield). EMA has been used with geospatial data to understand temporal and spatial associations between alcohol outlet density in communities and the incidence of specific alcohol-related problems (see the article by Freisthler). Technological advances in monitoring the drinking behavior of those who drive under the influence and other offenders (e.g., telemetry of alcohol sensor data and GPS tracking) aim to reduce recidivism (see the article by Voas). Beyond self-report and sensor data, real-time social media exchanges represent an understudied source of data on channels of peer and social influence relevant to understanding, for example, the spread of risky drinking behavior in a network (see the article by Moreno). These emerging eHealth technologies permit greater precision of measurement and more continuous monitoring of behavior in real time, with the possibility of simultaneous collection and integration of data from multiple sources (e.g., self-report, biosensor, social media) to better understand and predict behavior as it unfolds in naturally occurring contexts.

The capability for real-time data capture in natural contexts could transform theories of behavior change and models of health care delivery through personalized monitoring and just-in-time intervention (Riley et al. 2011). Traditional theories of behavior change are based on models of behavior sampled at relatively long intervals (e.g., weeks or months). In contrast, novel conceptual models based, for example, on dynamic systems theories (e.g., modeling dynamic feedback processes to maintain a steady state) applied to EMA data could offer important insights into the mechanisms of change in alcohol treatment (see the article by Morgenstern). An important goal of real-time monitoring is not only to predict the occurrence of risky behavior but to have just-in-time intervention when it may have maximum effectiveness (Lagoa et al. 2014). To realize the goal of just-in-time intervention, efficient study designs (Baker et al. 2014) and innovative statistical methods appropriate for analysis of “big data” (i.e., time intensive, multisource data) that involve, for example, machine learning and systems science applications, are indicated (Banks et al. 2014; Kumar et al. 2013; Timms et al. 2014).

In the translation to clinical practice, eHealth technologies have an important role as “clinician extenders” (e.g., adjuncts to formal treatment, support system during continuing care). Pioneering work to develop and evaluate an integrated eHealth system of delivery for alcohol treatment and recovery management (see the article by Quanbeck) holds promise for the use of technology to optimize doctor–patient communication and to sustain treatment gains over time. However, eHealth faces challenges in the translation to clinical practice. Such challenges include maintaining the user’s interest for extended periods of time through, for example, the use of persuasive technologies. These technologies are explicitly designed to change the user’s attitudes or behaviors during an electronic interaction using methods of persuasion or social influence (see the article by Muench). Another important consideration is the need to address the ethical issues in eHealth research and intervention delivery related to confidentiality and privacy (see the article by Arora). Perhaps the greatest challenge to widespread adoption of eHealth technologies involves the need for a new model of health care that integrates in-person and eHealth technologies across the continuum of care (Marsch 2012), and that explicitly acknowledges the usefulness of personal monitoring and dynamic, adaptive, interactive interventions delivered just in time to foster healthy behaviors (Riley et al. 2011).

eHealth is changing the way we conduct public health surveillance and conceptualize processes of behavior change. It also is catalyzing a transformation of health care by promoting new tools for personalized monitoring and intervention and creating new models for health care delivery. The exciting prospects for eHealth need to be considered in the context of ongoing challenges in bringing eHealth up to scale, optimizing the efficiency of monitoring and evaluating just-in-time intervention effectiveness, and addressing ethical concerns related to eHealth monitoring and application in clinical practice. Addressing these challenges will bring us closer to realizing the goals of improving our understanding of alcohol-related behaviors and preventing and treating alcohol use disorders.
